# Pediatric Psychology

**DOI:** 10.3390/pediatric13010020

**Published:** 2021-03-22

**Authors:** Giovanna Perricone

**Affiliations:** 1 Società Italiana di Psicologia Pediatrica (S.I.P.Ped), 90144 Palermo, Italy; giovanna.perricone@unipa.it; 2 Department of Psychology, Educational Science and Human Movement, University of Palermo, 90128 Palermo, Italy

The attention and the intervention of psychology in the field of pediatrics, especially regarding mothers and childcare, whether in a hospital or not, is extremely longstanding [1]. The intervention of psychology in the field of pediatrics and, more specifically, in maternal care and childcare, has been applied increasingly not simply to the clinical field but to the interface between psycho-social and clinical aspects. The conceptual field of pediatric psychology started to emerge when, in the 1960s, Jerome Kagan [2] promoted strong interaction and cooperation with pediatrics, thereby responding to the needs of pediatricians who found themselves confronted with psycho-developmental and behavioral problems in many children. This field was formalized in 1968 with the establishment of the Society of Pediatric Psychology, a society which was then to become a subdivision of the Division of Clinical Psychology (APA) and later gain scientific autonomy in the 54th division of the APA (2001).

In the years that followed, pediatric psychology also developed in Europe through the European Pediatric Psychology Network (EPPN). In Italy, the first contributions came from university research groups (Università degli Studi di Palermo, Università degli Studi di Padova, Università degli Studi di Genova, etc.) and hospital units (Unit of Pediatric Psychology of the Bambino Gesù Children’s Hospital). In this context, the know-how and expertise gave origin to the developmental psychology of a clinical-developmental matrix [3], creating an opportunity to respond to the lack of or too generalized psychological knowledge in the field of pediatrics, in terms of schools, theories, specific spaces and times, and an early concept of care.

The constructs of this know-how are certainly not new (i.e., work on managing resources); however, what is undoubtedly new is how single, interrelated constructs led to the creation of a model (the *know-how* of pediatric psychology) by integrating phenomenological knowledge, humanistic knowledge, knowledge “underlining the experientiality” [4] and cognitive constructivist knowledge [5].

Regarding expertise, a number of specific skills relating to the management of the know-how have allowed professional practice to go beyond psychology in pediatrics. Thus, the progression from applied psychology to expertise in pediatric psychology is attributed to the identification of the latter as a specific developmental theory, whereas, when speaking of psychology in pediatrics, places and conditions are often associated with a psychological clinic (psychopathology) rather than with a developmental theory. Furthermore, the vertex of this developmental theory is not psychodynamic nor clinical-developmental but a developmental-clinical vertex. Therefore, developmental processes form the object of study and intervention of the know-how defining pediatric psychology. These processes are analyzed and interpreted in all their developmental complexity; the clinical method is used to intervene [6], seeking help from a psychological clinic only after detecting a form of psychopathology and not *tout court*. Thus, such a change is not merely in name and also represents the idea that the clinical method (techniques, instruments, perspectives) allows intervention in analysis and in the developmental study of cognitive, relational and affective processes, etc. This study interprets its strengths and critical points and/or weaknesses, and the impairments often implicated in specific pathologies by intervening with the clinical method approach.

Within this know-how and expertise, the pediatric psychologist is no longer the health professional exclusively associated with anxiety and depression.

In addition to the constructs described here, there are criteria, perspectives, and approaches that identify and characterize pediatric psychology. Among these is the idea that health integrates biological, social, and psychological factors with moderating [7] and mediating variables. In this regard, there are a number of reference factors in developmental processes that facilitate conditions of wellbeing or dysfunctionality, weakness and distress. An example is found in the representations of a person’s physical health conditions, based on feelings of strength and resistance to life events. This idea of health calls to mind its consideration as a condition of wellbeing and identifies it as a particular meaning of mental health [8].

Health is presented according to an integrated method, which then crosses into the biopsychosocial field by considering the cognitive, affective, relational, and social variables in developmental processes. In pediatric conditions, health becomes a complex, dynamic condition determined by the result of managing the conflict between needs that are typical of development and special needs caused by the pathology, and also by the profile of the self being formed. Indeed, the nature of the complexity of self-perception becomes a moderating variable in states of distress based on the representative characteristics of the ideal self: how the child suffering from a disease *would like to be*; the real self: *how they represent themselves in the here and now of hospitalization*, and the fear of being or becoming, in a certain sense, inadequate, for example [9].

Such dynamics give rise to experiences that, if interiorized in a negative or regressive way, can lead to a dysfunctional vulnerability by creating risk conditions for the “inner space” and the “inner operative models” of the child/adolescent (c/a) [10,11].

Since health in a pediatric condition is a dynamic process, *turning points*—both those of a developmental nature and those constituting significant steps in the care path—seem to lead the quest for health, from the onset to the diagnosis, and subsequently to treatment and recovery or the “stabilization” of chronicity. Turning points of a developmental nature are characterized by the development of the agency [12] and, therefore, by the ability of the c/a to play a central role in cognitive process transformation (e.g., the transformation of the self-representative map) [13,14]. This transformation moves from a medical or medicalized map (indicating the elements of care, the care contexts and treatment) to a social map of beliefs and the social imaginary of that disease in childhood, and even towards an individual map, described *“as a personal construction that the child has developed over time in relation to the structures and functions of their own body, as well as to its external aspects, and their relationship with others and with the outside world; a mapping which leads to the representation of the sense of the child’s history and daily life”* [15].

Among the criteria, approaches, and perspectives, the resilience assumed by the context (e.g., treatment by the care system or during hospitalization) should be added to this representation of health. This means that the context in pediatric psychology should not be considered as a variable, but as an epiphenomenon of the health phenomenon [16]—that is, as an independent expression, made possible thanks to the conducts, roles, assumptions and activities carried out and unambiguously linked to the state of health. Indeed, if it is true that the context of pediatrics is defined independently through the roles and relationships that the c/a suffering from a pathology establishes with caregivers and health professionals, or activities in which the c/a is involved which are defined by the lack of wellbeing, it is also true that a condition collateral to this exists, as, in other health conditions, this context would have a different profile. However, the opposite is also very much true—i.e., that the context, with its specificity (in the systemic-ecological sense), orients the specific health condition [17].

Regarding the reference perspectives of pediatric psychology, the *strengthening* perspective [18,19] must be considered as a pillar, together with the management of resources, and as a priority strategy in intervention and its developmental-clinical vertex; the resilience of the child and their family is thus built [20].

The *strengthening* perspective [3,18,19] involves a sort of developmental result of stability and reinforcement of the developmental functioning [21,22] in terms of abilities and processes which become resources to focus on when working on developmental impairments and, therefore, building resilience. This building process involves the promotion of reparative processes activated by intervention. However, these are not exhaustive and have to be integrated with rehabilitative processes that allow for the search and enhancement/transformation of these resources.

*Strengthening* provides a real opportunity for risk coping. Indeed, it promotes creative adaptation [23,24,25], both by developing contact and problem-solving skills [26,27,28], by enhancing coping strategies [29,30] and promoting development of the child’s coping skills [31,32], which involve relational skills, such as knowing, classifying, and facing events (ibidem). Finally, the mobilization of self-protection mechanisms, which become self-defense mechanisms, has to be considered [32,33,34].

These protection mechanisms become the priority focus in the development of self-defense, on the one hand, as an object of investigation in managing the dynamics of development and, on the other hand, as the focus of an enhancement intervention, calling on noncognitive, social and identity construction processes, etc. In this sense, all the following processes can be involved: self-regulation processes; shared or nonsustained attention processes and attentional shift; all the steps of problem-solving; the definition of schemes and formats to manage the elements, situations, and relationships that the child perceives as “dangers”. Great importance has to be attributed to those representing the chance for the c/a to circumscribe their condition (the boundaries), thereby avoiding the intrusion of any other (possibly destructive) element and specific consequence. The child may assess these as obstacles to the “catastrophic” change [35], intended as a cognitive and not just an emotional element functional to learning, which may destroy, for example, a positive model of the self. This element is presented as an unavoidable change, which, as such, despite apparently destroying the c/a’s certainties, allows them to find other possibilities, starting from the specific condition.

The “boundary”, defined by the child’s opportunity to circumscribe their condition, is functional to loneliness, not as a state of isolation, but as a strategic withdrawal oriented to accept the destructive element of the disease, by giving this element the possibility to imply, recall and create the basis for a functional change.

Exploration of the resources referred to in pediatric psychology is proprietarily limited to specific fields, such as social and cognitive processes, etc.

The perspectives and criteria indicated are the connective tissue of the know-how constructs of pediatric psychology. Among these, three are the fundamental constructs in the interpretation of development, the object of study and intervention of pediatric psychology: the developmental trajectory, the developmental energy, and the dynamics of development [36,37,38,39,40].

The *developmental trajectory*, which replaces the developmental pathway idea, is defined as a development trend which, being oriented by specific biological, epigenetic and cultural factors, occurs via interaction between the evolvement of the different dimensions (social, cognitive, etc.) and the mentalization of the experience of disease, cure, etc., which, instead, lays down the developmental trajectory.

The *developmental energy*, which crosses the trajectory and is the engine of the dynamics of development, results from systemic brain production, which gives rise to the network of synchronic functioning responsible for developmental orientation.

The *dynamics of development* [41], set in motion by the developmental energy underlying the trajectory, develop through a balance between development diverging trends (continuity/discontinuity, nature/nurture, etc.) and orient the dysfunctionality of cognitive and relational processes shown through maladaptive conducts in the child suffering from a pathology. These maladaptive conducts serve as deterrents to functional processes by hindering, for example, forms of preparatory alliances in the management of the pathology between the child and the family and the child and the care system.

The construct of *pediatric condition* intends to replace the idea of considering a child suffering from a pathology as a “case” by acknowledging a real pediatric condition—i.e., a field of relations between single forces [42,43,44].

Pediatric psychology defines this condition of a psychological state as the specific plot of the relationship between single “forces” of a “field” [3,36]. This field thus develops from the relationships originating from the child regarding: psychological and organic resources; organic impairments and their related severity; weaknesses; the type of developmental crisis generated by the disease seen as a critical event; any disability; the nosologic framework; prospects of cure; the quality of life perceived [45,46]. Other forces are identified in the relationships originating from the child and directed to other systems: the family and their subsystems; the parental couple and their levels of cohesion; the type of parenting skills and the possible dysregulations on which a parental style is developed; the parental couple’s ability to creatively adapt to the situation; the prediction of the future and, therefore, planning for their children; the type of representation of the condition of the disease; any anxiety about uncertainties; phratry as a possible present resource [47], and the family’s allostasis or homeostasis [48]. Among the forces, the care system and its medical and clinical interventions, its operators, the organization between professionals, the position, the localization and/or delocalization of the psychologist, any integrated work between these professionals, any path of accompaniment to hospital, home accompaniment, and local services must be taken into consideration.

Other reference forces may exist; in any case, the field is defined via the relationships that the c/a in their uniqueness has with the family, contexts, and care system. *“The relationships with the single forces constitute, by nature, the c/a’s world of more or less evident, more or less significant bonds. It is within these bonds that the potential resources, the functional or dysfunctional development of resilience, but first and foremost, the self and the way the c/a approaches the disease are defined, with a significant contribution from the representations, affections, arousal mechanisms, self-perception etc.”* [17].

The definition of the pediatric condition leads to its identification as a developmental emergency taking shape from the relation between the child’s impairments and resources.

Working on trauma is one of its constructs. Trauma consists of a series of emotional experiences linked to particularly distressing, anguishing, intolerable moments, phases, and situations related to the disease and, therefore, mentalized as a negative experience and perceived as so deconstructing that even the thought of it cannot be tolerated [49]. These conditions are caused by a traumatic factor (stressor) which may have psychopathological results, such as dissociative disorders or the disorganization of integrated functions of consciousness, memory, identity, perception and representation of the environment [50,51,52].

Any pediatric condition often has to deal with trauma, which may occur at the onset, the diagnosis, or after a succession of microtraumas linked to treatments. In pediatric psychology, trauma must be identified as the background and origin of emotional resonances induced by the impairment and the alteration of the dynamics of development. It is managed as a relapse of work on the dynamics of development, aiming to build a new world of active emotional resonances. These resonances may develop a new “inner space” functional to redefine the traumatic condition, starting from making the event that determined it as “thinkable”.

In pediatric psychology, the trauma condition in the developmental trajectory is an interruption of a typical continuity “felt” by the child, and it is placed between the mentalization (in a deconstructing sense) of the experiences of disease, care, treatment, etc., and the intentionality related to the possibility of managing these experiences. This interruption makes it impossible to give a sense and a meaning to the disease event, which is functional at a developmental level. Perhaps this interpretation of trauma precisely guides us to define the overall aim of the know-how and expertise in pediatric psychology. This must consist of giving meaning, not only to and not as much to the state of disease, but to the pediatric condition, and, therefore, to the state of weakness and inadequacy felt by the c/a, and also the relationship with parents, etc.

All these main constructs of pediatric psychology identify the condition of the child suffering from a pathology as a risk condition which may alter self-esteem, and compromise or slow down a number of processes, often induced by treatments. A particularly illustrative example is given by impairments which occur in the conditions of developmental crisis caused by tumors. The following impairments should be mentioned: slowing of motor development [53]; dysfunctionality of emotional development, depression; alteration of self-esteem [54,55]; feelings of loss [3]; the correlation between coping/monitoring strategies and perceived parental stress [56]; hyper control and anxiety levels [57].

*“When a different know-how is defined within the scientific tradition of a field, a wind seems to rise which, depending on the transformative force of this know-how and the social and political “interests” of the category, can be felt as a shattering force or like a breeze that brings perturbations. This seems to be the destiny, designated by the Community of Psychologists for Pediatric Psychology, when there is change from an applicative and generalizable form to specific know-how and expertise which circumscribe, distinguish, overturn and differentiate”* [17].
